# Relationship Between Sarcopenia and Prognosis in Patient With Concurrent Chemo-Radiation Therapy for Esophageal Cancer

**DOI:** 10.3389/fonc.2019.00366

**Published:** 2019-05-08

**Authors:** Dae Won Ma, Yeona Cho, Mi-jin Jeon, Jie-Hyun Kim, Ik Jae Lee, Young Hoon Youn, Jae Jun Park, Da Hyun Jung, Hyojin Park, Chang Geol Lee, Jun Won Kim, Hei Cheul Jeung

**Affiliations:** ^1^Department of Internal Medicine, Yonsei University College of Medicine, Seoul, South Korea; ^2^Gangnam Severance Hospital, Yonsei University College of Medicine, Seoul, South Korea; ^3^Department of Radiation Oncology, Yonsei University College of Medicine, Seoul, South Korea; ^4^Severance Hospital, Yonsei University College of Medicine, Seoul, South Korea

**Keywords:** esophageal cancer, concurrent chemo- and radiotherapy, sarcopenia, prognosis, complication

## Abstract

**Background:** Sarcopenia, defined as skeletal muscle loss, has been known as a poor prognosis factor in various malignant diseases The aim of this study is to investigate the effect of sarcopenia on prognosis in patients with esophageal cancer who received concurrent chemo- and radiotherapy (CCRT).

**Methods:** We retrospectively collected clinical data of 287 patients with esophageal cancer who were treated by definite CCRT at Gangnam Severance and Severance hospital from August 2005 to December 2014. The cross-sectional area of muscle at the level of the third lumbar vertebra was measured using pre- and post-CCRT computed tomography images. Sarcopenia was defined as skeletal muscle index <49 cm^2^/m^2^ for men and of <31 cm^2^/m^2^ for women by Korean-specific cutoffs. Overall survival (OS) and progression free survival (PFS) were analyzed according to sarcopenia.

**Results:** Sarcopenia identified before CCRT did not affect OS and PFS. However, patients with post-CCRT sarcopenia showed shorter OS and PFS than patients without it (median OS: 73 months vs. 28 months; median PFS: 34 months vs. 25 months, respectively). Post-CCRT sarcopenia was an independent prognostic factor of poor OS (hazards ratio: 1.697; 95% confidence interval: 1.036–2.780; *P* = 0.036). In multivariate analysis, male sex (*P* = 0.004) and presence of CCRT-related complications, such as esophagitis or general weakness were significantly associated with post-CCRT sarcopenia (*P* = 0.016).

**Conclusions:** Sarcopenia after CCRT can be a useful predictor for long-term prognosis in patients with esophageal cancer. To control CCRT-related complications may be important to prevent skeletal muscle loss during CCRT.

## Introduction

Esophageal cancer is widely known as fatal and aggressive disease (1). In the past, surgical treatment was the standard treatment for esophageal cancer. Because of the poor prognosis after surgery, a multidisciplinary approach is being made for the treatment of esophageal cancer (2, 3). Concurrent chemo- and radiotherapy (CCRT) is not only a treatment option for inoperable esophageal cancer, but also is known as prefer approach for locally advanced esophageal cancer (4, 5).

The patients with esophageal cancer mostly suffer from dysphagia and poor oral intake, and it makes deleterious effects to treatment compliance and outcomes. Therefore, it is necessary for patients with esophageal cancer to help prevent malnutrition through adequate nutritional support (6). Sarcopenia is a condition characterized by loss of muscle mass and strength, and critically associated with numerous clinical conditions such as malnutrition, endocrine disease, inflammatory disease, and malignancy (7, 8). It was originally described in the elderly people, but now usually recognized in cancer patients. The decrease in muscle mass is not always correlated with changes in body weight, so it is not always the same as the decrease in body mass index (BMI). Recent studies suggest that changes in nutritional status and body composition are associated with prognosis in cancer patients (9–12).

To assess muscle mass, various measurement methods are used. Among them, computed tomography (CT) scan showed superiority in accuracy and precision for evaluating muscle and fat mass (13). Several studies have revealed the relationship between preoperative sarcopenia and prognosis in patients with esophageal cancer (14–17).

However, in our best knowledge, there is no study of the relationship between sarcopenia and prognosis in esophageal cancer patients who received definite CCRT. Therefore, we aimed to investigate the changes in body composition before and after CCRT and the impact of pre/post-CCRT sarcopenia on the prognosis of these patients.

## Materials and Methods

### Patients and Clinical Characteristics

All consecutive patients who were diagnosed with esophageal cancer and treated with definite CCRT at Gangnam Severance and Severance hospital from August 2005 to December 2014 were retrospectively collected for this analysis. A total of 287 patients with pathologically proven esophageal cancer were included after dedicated electronic medical records review. Of these, 9 patients had an additional surgery after CCRT, 25 patients had overlapping of esophageal cancer with another cancer, and 55 patients did not have sufficient CT images to measure muscle mass. Thus, except for these patients, a total of 198 patients were finally included for our study.

The initial performance status of patients was recorded by Eastern Cooperative Oncology Group (ECOG) score. Hemoglobin, albumin and neutrophil/lymphocyte ratio (NLR) at the time of diagnosis of esophageal cancer were collected to identify patient's nutritional and inflammatory status related to prognosis (18). Tumor location, histopathologic grade, clinical stage were classified according to AJCC/UICC staging (19). Esophagogastroduodenoscopy, CT, endoscopic ultrasound, and positron emission tomography-computed tomography (PET-CT) were applied to determine the clinical stage. Patients were observed at 3 to 6 months intervals until Aug 31, 2017 or death, whichever came first. Overall survival (OS) was defined as the length of time from the date of initial diagnosis to the date of death. Progression free survival (PFS) was defined as the length of time after treatment for a cancer ended that the patient survived without any signs of symptoms of the cancer. The Institutional Review Board of Gangnam Severance Hospital waived the need for approval of this study.

Radiotherapy (RT) was performed with 3-dimentional conformal RT or intensity-modulated RT (IMRT) starting on day 1 of chemotherapy and a conventional fractionation schedule (1.8–2.0 Gy per fraction, 5 days per week) and cone-down technique were used in all patients. The gross tumor volume (GTV) was delineated using (PEC/CT) fusion on the MIM software (Cleveland, OH) or Pinnacle Radiotherapy Planning System (Phillips Medical System, Andover, MA). Endoscopic clips were also used for contouring GTV. The initial clinical target volume (CTV) included the GTV plus a margin of at least 5 cm longitudinally and 2 cm radially. The initial CTV received 30.6–50.4 Gy (median dose, 36 Gy), and at the time of cone-down, final CTV encompassed the GTV with a 2 cm margin longitudinally and radially. The total prescribed radiation dose ranged from 50.4 to 66 Gy according to the physicians' decisions. Previous study in our institution reported that higher dose (>60 Gy) yielded better outcome without increase of any toxicity and higher dose scheme has been used in our institution (20).

Two cycles of chemotherapy were administered during CCRT period. Consolidation chemotherapy was conducted between 4 and 6 weeks after the completion of CCRT. Complications associated with CCRT were defined as development of 1 or more adverse effect with common toxicity criteria grade 2 or higher during CCRT period.

### Image Analysis

Because dual-energy X-ray absorptiometry (DXA) is used infrequently in routine clinical practice, we used a previously validated CT-based body composition method with scans acquired at the initial diagnosis (e.g., whole-body PET-CT or abdominal CT imaging). We also obtained post CCRT images that were taken within 6 months after the end of CCRT. The third lumbar vertebra (L3) was selected as a landmark for calculation since the cross-sectional area of tissues in this region provide an established means of estimating body composition parameters quantities in general population (21).

**Figure 1:**
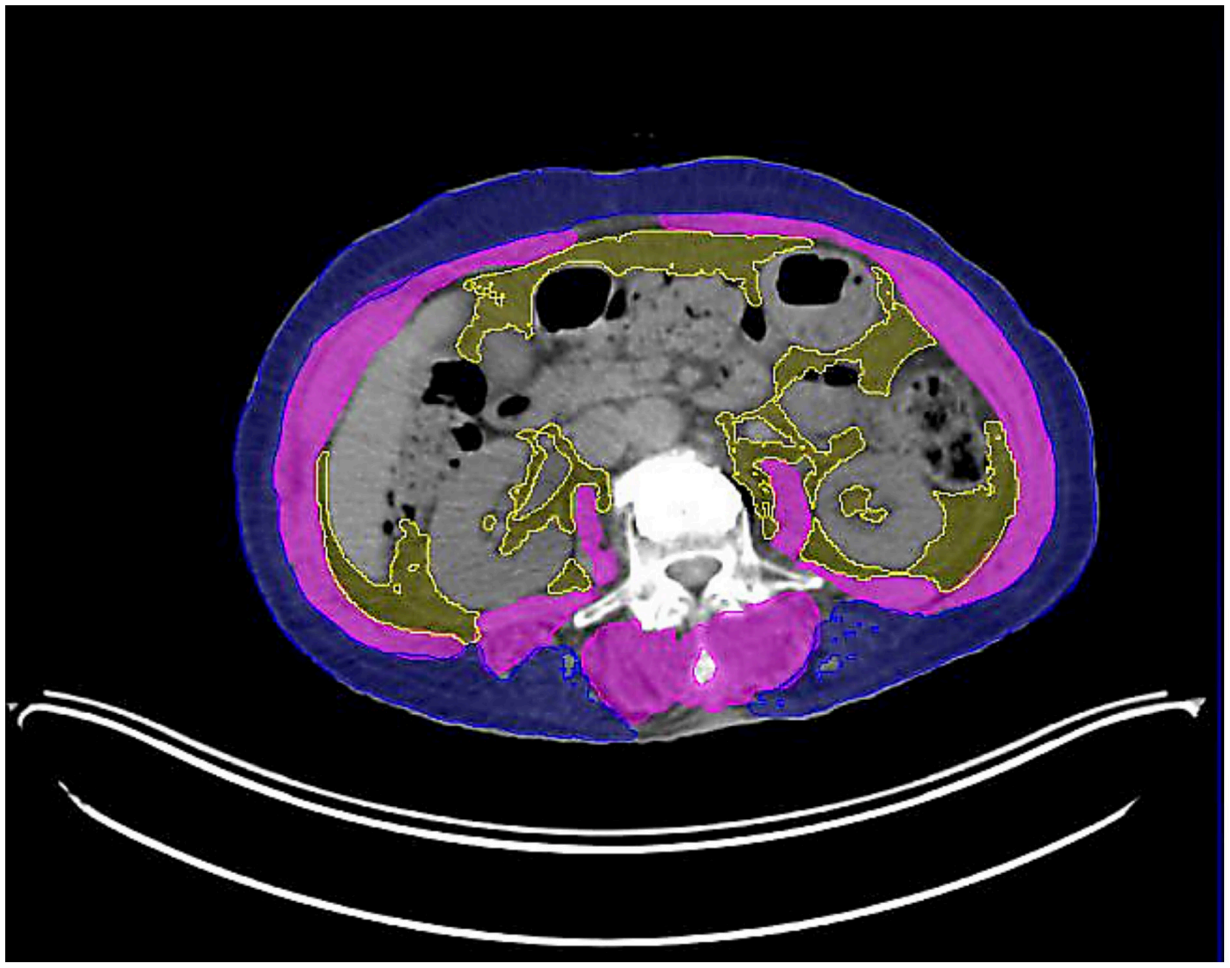
Axial computed tomography image of the third lumbar vertebra used for the assessment of skeletal muscle area (highlighted in pink).

We obtained skeletal muscle volume and total adipose tissue volume at L3 level, and divided by CT slice thickness, respectively, to calculate skeletal muscle area and total adipose tissue area. The MIM Vista software was used to demarcate skeletal muscle, visceral fat tissue, and subcutaneous fat tissue according to predefined validated boundaries based on Hounsfield units (HUs). The following thresholds were applied: −29 to +150 HU for skeletal muscle, −150 to −50 HU for visceral fat tissue, and −190 to −30 HU for subcutaneous fat tissue. The radiation oncologist and the radiotherapy technician (Ik Jae Lee and Mi-jin Jeon) who performed these measurements was blinded to the treatment outcomes of all patients to minimize bias (Figure 1).

Skeletal muscle index (SMI), fat-free mass (FFM), and total body fat mass (FM) were estimated as follows (21): SMI (cm^2^/m^2^) = skeletal muscle area (cm^2^) / height^2^ (m^2^), FFM (kg) = 0.3 ^*^ skeletal muscle area (cm^2^) + 6.06, FM (kg) = 0.042 ^*^ total adipose tissue area (cm^2^) + 11.2. In general, sarcopenia is defined as a L3 SMI of <55 cm^2^/m^2^ for men and <39 cm^2^/m^2^ for women, as proposed by international consensus of cancer cachexia (22). However, we used Korean-specific cutoff values of 49 cm^2^/m^2^ for men and 31 cm^2^/m^2^ for women based on a previous epidemiologic study using DXA and a regression equation,

$$L3\;muscle\;index\;of\;CT\;=\frac{\begin{array}{l} height-adjusted\;appendicular\;skeletal\\ muscle\;mass\;in\;DXA\;(kg/m2)-1.17 \end{array}}{0.11}$$

to convert the CT value (9).

### Statistical Analysis

All continuous variables were reported as median and range. The chi-square and Fisher's exact tests were used to compare between various categorical variables, and Student *t*-test and Mann-Whitney *U*-test were used for non-categorical variables. Multivariate logistic regression analysis was used to identify independent risk factors for predicting post–CCRT sarcopenia. OS and PFS were estimated using the Kaplan-Meier method, and differences between curves were evaluated using the log-rank test. A Cox proportional hazards model was used to analyze risk factors that affect OS. All variables with *P* < 0.05 was on the univariate analysis were entered the multivariate analysis. The statistical calculations were performed using SPSS version 18.0 for Windows software (SPSS Inc., Chicago, IL, USA).

## Results

### Baseline Characteristics of Patients and Tumors

A total of 198 consecutive patients were enrolled in our study (Table 1). The median age was 67 years (range 36–91), and most of the patients were men (96%). One hundred ninety patients (96%) had good ECOG performance score (0–1). Of the 198 patients, 46 (23.2%) patients underwent esophageal stent insertion due to obstructive symptoms. More than half (55.1%) of the tumors were in the middle esophagus. The histologic type of tumor was squamous cell carcinoma in most cases (98.5%) and adenocarcinoma in 3 cases. Of all patients, 67.2% had a clinical tumor stage greater than T2, and 74.2% had a clinical nodal stage greater than N0. According to clinical TNM stage, stage I was 30 patients (15.2%), stage II was 40 patients (20.2%), stage III was 68 patients (34.3%), stage IVA was 37 patients (18.7%), and stage IVB was 23 patients (11.6%). Median total radiation dose was 6,300 cGy. There were 140 patients (70.7%) who received the consolidation chemotherapy after CCRT. We found 66 patients (33.3%) who suffered from CCRT related complications. Among them, esophagitis was the most common (*n* = 48). Other complications were general weakness (*n* = 9), neutropenia (*n* = 6), pneumonia (*n* = 2), and tracheo-esophageal fistula (*n* = 1).

**Table 1:** Baseline characteristics of patients and tumors.

**Characteristics**	**All (*n* = 198)**
Age (years, median [range])	67 (36–91)
**Sex**
Male	190 (96.0)
Female	8 (4.0)
**ECOG performance status**
0–1	190 (96.0)
2–3	8 (4.0)
BMI (kg/m^2^, median [range])	22.6 (15.1–33.3)
Hemoglobin (g/dL, median [range])	13.6 (8.2–17.6)
Albumin (g/dL, median [range])	4.2 (2.7–5.1)
Neutrophil / lymphocyte ratio	2.3 (0.9–44.8)
**Esophageal stent insertion**
No	152 (76.8)
Yes	46 (23.2)
**Tumor location**
Cervical	13 (6.6)
Upper thoracic	42 (21.2)
Mid thoracic	109 (55.1)
Lower thoracic	34 (17.2)
**Tumor histology**
Squamous cell carcinoma	195 (98.5)
Adenocarcinoma	3 (1.5)
**Tumor differentiation**
Well differentiation	25 (12.6)
Moderate differentiation	113 (57.1)
Poorly differentiation	41 (20.7)
Gx	19 (9.6)
**Clinical tumor stage**
T1	34 (17.2)
T2	31 (15.7)
T3	103 (52.0)
T4	30 (15.2)
**Clinical nodal stage**
N0	51 (25.8)
N1	107 (54.0)
N2	22 (11.1)
N3	18 (9.1)
**Chemotherapy regimen**
5FU + Cisplatin	191 (96.5)
Others	7 (3.5)
Radiation dose (cGy, median [range])	6300 (3060–7020)
**Consolidation chemotherapy**
No	58 (29.3)
Yes	140 (70.7)
CCRT complication^*^
No	132 (66.7)
Yes	66 (33.3)

*ECOG, Eastern Cooperative Oncology Group; BMI, body mass index; Gx, grade cannot be assessed; CCRT, concurrent chemo- and radiotherapy*.

* *Esophagitis, general weakness, neutropenia, pneumonia, tracheo-esophageal fistula*.

### Changes in Body Composition Before and After Treatment

We compared BMI, SMI, FFM, and FM before and after CCRT except 25 patients who did not have post–CCRT CT image. BMI, SMI, and FFM after CCRT were statistically significantly decreased (*P* < 0.001). Also, we identified 25 patients with sarcopenia following CCRT. However, FM did not show any significant difference before and after CCRT. In female subgroup, there was no statistically difference in BMI before and after CCRT (Table 2).

**Table 2:** Comparison of changes in body composition before and after CCRT.

**Parameters**	**Before CCRT**	**After CCRT**	***P*-value**
**ALL**			
Body mass index (kg/m^2^)	22.6 (15.1–31.2)	21.5 (13.3–30.7)	< 0.001
Skeletal muscle index (cm^2^/m^2^)	46.0 (27.2–67.7)	42.2 (20.0–81.4)	< 0.001
Fat free mass (kg)	44.5 (29.1–63.2)	41.5 (21.3–75.8)	< 0.001
Total body fat mass (kg)	18.9 (11.4–30.4)	18.2 (11.3–28.6)	0.207
**MALE**			
Body mass index (kg/m^2^)	22.6 (15.1–31.2)	21.4 (13.3–30.7)	< 0.001
Skeletal muscle index (cm^2^/m^2^)	46.3 (27.2–67.7)	42.6 (20.0–81.4)	< 0.001
Fat free mass (kg)	44.9 (29.0–63.2)	41.6 (21.3–75.8)	< 0.001
Total body fat mass (kg)	18.8 (11.4–30.4)	18.1 (11.3–28.6)	0.345
**FEMALE**			
Body mass index (kg/m^2^)	23.5 (17.0–28.0)	22.6 (16.6–27.7)	0.123
Skeletal muscle index (cm^2^/m^2^)	38.4 (31.3–48.7)	33.8 (26.0–38.8)	0.012
Fat free mass (kg)	33.1 (30.2–38.1)	28.7 (25.0–32.8)	0.012
Total body fat mass (kg)	22.7 (14.2–26.3)	20.5 (14.4–26.1)	0.263

*CCRT, concurrent chemo- and radiotherapy*.

### Analysis of Risk Factors Affecting Overall Survival Rate

**Figure 2:**
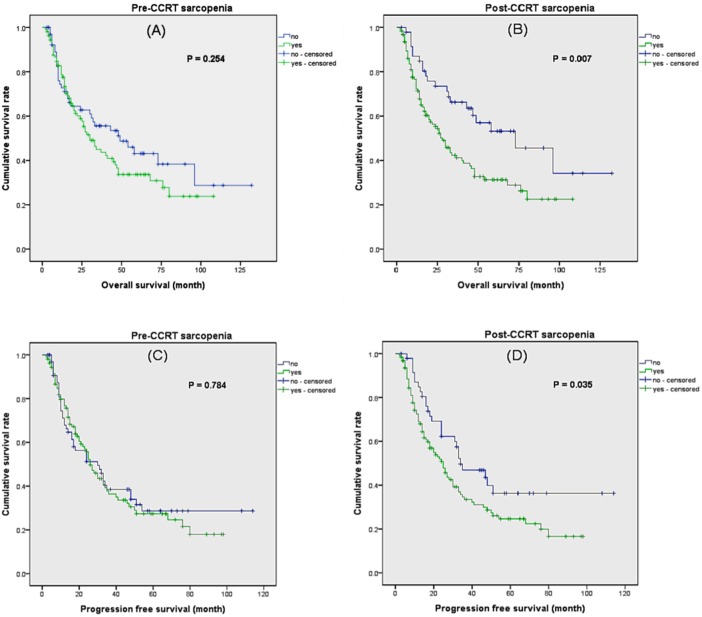
Kaplan–Meier analysis of overall survival and progression free survival according to sarcopenia. **(A)** Overall survival according to pre–CCRT sarcopenia. **(B)** Overall survival according to post–CCRT sarcopenia. **(C)** Progression free survival according to pre–CCRT sarcopenia. **(D)** Progression free survival according to post–CCRT sarcopenia.

Patients with sarcopenia before CCRT did not showed significantly decreased OS compared to non-sarcopenic patients (Figure 2). However, patients with sarcopenia after CCRT demonstrated significantly poor OS compared to non-sarcopenic patients (median OS 45 months vs. 74 months, *P* = 0.007). Also, patient with sarcopenia after CCRT showed poor PFS compared to non-sarcopenia patients, but patients with sarcopenia before CCRT did not.

We analyzed several risk factors that might influence OS (Table 3). In univariate analysis, higher ECOG performance status (≥2), higher NLR, clinical tumor stage (≥cT3), and clinical nodal stage (≥cN1) showed significant relationship with OS. The presence of sarcopenia before CCRT did not affect OS. In contrast, patients who were diagnosed as sarcopenia after CCRT showed impaired survival. Multivariate analysis revealed that higher ECOG performance status, higher NLR, higher clinical tumor stage, and post-CCRT sarcopenia were independent risk factors for decreased OS. Furthermore, SMI decrement was associated with poor OS regardless of sarcopenia. On the other hand, tumor location, tumor differentiation, radiation dose, consolidation therapy, and CCRT complication did not show statistical correlation with OS.

**Table 3:** Univariate and multivariate analysis of risk factors for overall survival.

	**Univariate analysis**		**Multivariate analysis**	
	**HR (95% CI)**	***P*–value**	**HR (95% CI)**	***P*–value**
Age	0.994 (0.971–1.017)	0.598		
Sex (male)	2.059 (0.507–8.369)	0.313		
ECOG performance status (≥ 2)	5.195 (2.247–12.008)	0.001	3.805 (1.605–9.023)	0.002
NLR	1.065 (1.025–1.107)	0.001	1.059 (1.018–1.102)	0.005
Tumor location (middle & lower)	1.135 (0.719–1.793)	0.587		
Tumor differentiation	1.076 (0.637–1.819)	0.784		
cT stage (≥ cT3)	1.872 (1.197–2.927)	0.006	1.586 (1.004–2.504)	0.048
cN stage (≥ cN1)	1.842 (1.090–3.113)	0.023	1.354 (0.779–2.352)	0.283
Radiation dose (< 50Gy)	1.862 (0.861–4.029)	0.114		
Consolidation therapy (no)	1.121 (0.714–1.760)	0.619		
CCRT complication (yes)	1.216 (0.803–1.841)	0.355		
Pre-CCRT BMI (< 23 kg/m^2^)	1.417 (0.944–2.126)	0.093		
Post-CCRT BMI (< 23 kg/m^2^)	1.431 (0.910–2.251)	0.121		
BMI decrement	1.058 (0.955–1.173)	0.278		
Pre-CCRT sarcopenia	1.273 (0.837–1.937)	0.254		
Post-CCRT sarcopenia	1.923 (1.183–3.127)	0.007	1.651 (1.007–2.704)	0.047
SMI decrement	1.049 (1.011–1.088)	0.011	1.042 (1.001–1.086)	0.046

*HR, hazard ratio; ECOG, Eastern Cooperative Oncology Group; NLR, Neutrophil / lymphocyte ratio; cT, clinical tumor; cN, clinical nodal; CCRT, concurrent chemo- and radiotherapy; BMI, body mass index; SMI, skeletal muscle index*.

Also, we analyzed what factors affected post–CCRT sarcopenia. The relationship between the factors described in Table 1 and post–CCRT sarcopenia was analyzed. As a result, male sex, CCRT complications, and tumor differentiation (moderate and poorly differentiation) showed statistically significant relationship in univariate analysis. In multivariate analysis, male sex, and CCRT complications were significantly associated with post–CCRT sarcopenia (Table 4).

**Table 4:** Multivariate analysis of risk factor for post-CCRT sarcopenia.

**Characteristics**	**Odd ratio (95% CI)**	***P*-value**
Sex (male)	11.967 (2.060–69.520)	0.006
CCRT complication^*^	2.741 (1.169–6.425)	0.020
Tumor differentiation (MD or PD)	2.625 (0.930–7.410)	0.068

*CCRT, concurrent chemo- and radiotherapy; MD, moderate differentiation; PD, poorly differentiation*.

* *Esophagitis, general weakness, neutropenia, pneumonia, tracheo-esophageal fistula*.

### Changes in Sarcopenic Status After CCRT and Overall Survival

**Figure 3:**
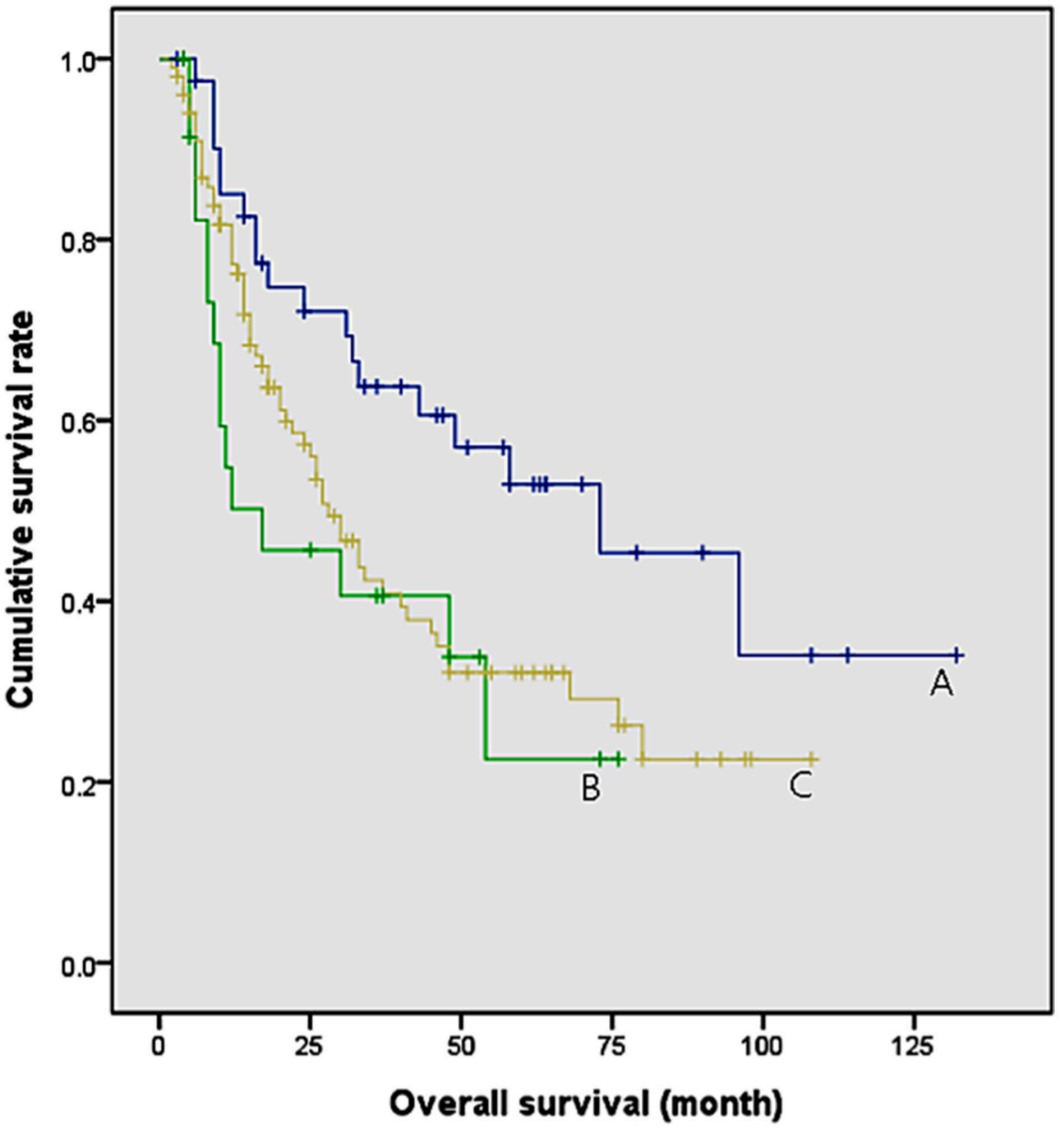
Comparison of survival rates according to the time of onset of sarcopenia. Group A: patients who were not diagnosed with sarcopenia after CCRT (median survival = 73 months). Group B: patients who were not diagnosed with sarcopenia before CCRT, but were diagnosed with sarcopenia after CCRT (median survival = 17 months). Group C: patients who were diagnosed with sarcopenia before and after CCRT (median survival = 28 months). Group A and B, *P* = 0.016; Group A and C, *P* = 0.024; Group B and C, *P* = 0.454.

All study population were classified according to the timing of onset of sarcopenia (Figure 3). They were divided 3 groups: patients who were not diagnosed with sarcopenia after CCRT (group A, *n* = 48, median follow up = 38 months), patients who were not diagnosed with sarcopenia before CCRT, but were diagnosed with sarcopenia after CCRT (group B, *n* = 25, median follow up = 11 months), patients who were diagnosed with sarcopenia before and after CCRT (group C, *n* = 101, median follow up = 20 months). Patients who had never been diagnosed with sarcopenia (group A) had a significantly longer survival rate than group B and C (*P* = 0.016 and 0.024, respectively). However, patients with post–CCRT sarcopenia had no difference in survival rates regardless of pre–CCRT sarcopenia (*P* = 0.454). In other words, patients with post–CCRT sarcopenia showed worse overall survival than patients without post–CCRT sarcopenia. Furthermore, the presence of pre–CCRT sarcopenia did not affect the overall survival rate.

## Discussion

In this study, we investigated the relationship between the sarcopenia and the prognosis of patients with esophageal cancer who treated with definite CCRT. Although there have been many similar studies, most of them have been related to surgery, and studies in patients with CCRT have been lacking (14–17). In addition, our study is characterized by comparing muscle mass before and after treatment. As a result, the patient with sarcopenia before CCRT did not show significant association with overall survival, but the patient with sarcopenia after CCRT did. In other words, presence of sarcopenia after CCRT affected an unfavorable effect on the prognosis, regardless of whether it occurred before or after treatment. Also, it was found that male sex and CCRT related complications were associated with post CCRT sarcopenia.

The relationship between sarcopenia and prognosis has been known as controversial in patients with surgically treated esophageal cancer. Sheetz et al. reported that core muscle size was associated with poor OS, in patients following esophagectomy. In contrast, it was not associated with OS, in patients receiving neoadjuvant chemoradiation (6). Similar to this study, another study found that the presence of sarcopenia did not show significant correlation with a short- and long-term outcome in esophageal cancer patients after neoadjuvant chemoradiotherapy followed by esophagectomy (14). However, there were several papers that proved the relationship between sarcopenia and clinical outcome in patients after esophageal resection who underwent neoadjuvant chemoradiotherapy (15–17). Our study shows differences from previous studies in that only patients who underwent definite CCRT were included. In addition, the previous studies were mainly executed in Western countries such as Europe and the United States, but our study is characterized by research conducted in Asia where esophageal squamous cell carcinomas develop more often than adenocarcinoma.

Sarcopenia could be a characteristic of esophageal cancer itself or one of the complications associated with treatment. Cancer treatments such as surgery, chemotherapy, or RT are known to contribute to muscle loss by causing anorexia. Also, various circulating inflammatory mediators such as tumor necrosis factor-α and interluekin-6 have been implicated in excessive muscle proteolysis (23). In our study, we revealed that patients with sarcopenia after CCRT were associated with a poor prognosis. In contrast, the presence of sarcopenia before treatment was not associated with prognosis. CCRT complications were one of the factors when multivariate analysis of factors related to the incidence of post-CCRT sarcopenia. Although the cause of sarcopenia after CCRT is not yet established, the characteristics of esophageal cancer itself, and changes in nutritional status due to the complications of CCRT may contribute to sarcopenia. In our study, esophagitis was the most common complication after CCRT. It could cause dysphagia or odynophagia, and aggravate malnutrition for patients (24). Not only topical anesthetics and analgesics, but also dietary modification could be helpful to control the symptoms (25). Preventing the occurrence of sarcopenia by providing adequate nutritional support and proper management for complications during the CCRT period could facilitate the improvement of the patient's prognosis.

Our study has several limitations. First, we collected data retrospectively from electro medical records. It was not a prospective study, and some data were limited. We could not include 25 patients who did not undergo skeletal muscle mass assessment after CCRT, and they should be considered in the interpretation of data because the possibility of their poor prognosis. Also, toxicity surveillance was performed imperfectly due to retrospective design. Second, the definition of sarcopenia was different from that of Western countries. International consensus of cancer cachexia proposed the definition of sarcopenia as a L3 muscle index of <55 cm^2^/m^2^ for men and of <39 cm^2^/m^2^ for women (22). However, we did not use this definition because the BMI of Western and Eastern people were quite different. If western definitions are applied, the incidence of pre-CCRT sarcopenia in our study rises from 62.6 to 84.8%. We consider that the definition used in Western countries are not necessarily applicable to Korean esophageal cancer patients who generally have a smaller physique.

In conclusion, we investigated the relationship between prognosis and sarcopenia in esophageal cancer patients receiving definite CCRT, and found that patients with sarcopenia identified after CCRT were associated with a poor prognosis. That is to say, post–CCRT sarcopenia can be a reliable predictor for prognosis. This means that proper support for preventing skeletal muscle loss during CCRT may help improve the prognosis of patients with esophageal cancer. In particular, complications that occur during CCRT may impair the nutritional status of the patient, so it is important to cope with complications appropriately.

## Ethics Statement

The Institutional Review Board of Gangnam Severance Hospital waived the need for approval of this study.

## Author Contributions

DWM conducted the analysis and interpretation of the data, drafted the article, and revised it critically for important intellectual content. YNC made substantial contributions to the analysis and interpretation of the data, and revised it critically for important intellectual content. M-JJ made substantial contributions to the analysis and interpretation of the data. J-HK made substantial contributions to the conception and design, interpreted the data and revised it critically for important intellectual content, approved the article for publication and agreed to be accountable for all aspects of the work. IJL interpreted the data, critically revised it for important intellectual content, approved the article for publication and agreed to be accountable for all aspects of the work. YHY, JJP, DHJ, HJP, CGL, JWK, and HCJ critically revised the article for important intellectual content.

### Conflict of Interest Statement

The authors declare that the research was conducted in the absence of any commercial or financial relationships that could be construed as a potential conflict of interest.
